# A Lightweight ScaleDense–Transformer Framework with Auxiliary Quantum-Inspired Bottleneck Module for Whole-Lifespan Brain Age Prediction

**DOI:** 10.3390/brainsci16060581

**Published:** 2026-05-29

**Authors:** Lan Lin, Xinyu Zhu, Hongjian Gao, Shen Sun

**Affiliations:** 1 Department of Biomedical Engineering, College of Chemistry and Life Science, Beijing University of Technology, Beijing 100124, China; lanlin@bjut.edu.cn (L.L.); zhuxyu@emails.bjut.edu.cn (X.Z.); gaohongjian@bjut.edu.cn (H.G.); 2 Intelligent Physiological Measurement and Clinical Translation, Beijing International Base for Scientific and Technological Cooperation, Beijing University of Technology, Beijing 100124, China

**Keywords:** brain age prediction, deep learning, neuroimaging, ScaleDense–Transformer, quantum-inspired architectures

## Abstract

**Highlights:**

**What are the main findings?**

- The proposed framework achieves a state-of-the-art Mean Absolute Error (MAE) of 2.71 years in whole-lifespan brain age prediction (0–96 years), comparing favorably with standalone CNN and Transformer baselines.
- LST-Net demonstrates consistent cross-cohort validation boundaries and maintains reproducible prediction accuracy across multi-site neuroimaging repositories, yielding a stable test–retest reliability (ICC = 0.994) and high longitudinal consistency.

**What are the implications of the main findings?**

- LST-Net provides a scalable and biologically plausible tool for large-scale brain health monitoring, enabling precise tracking of neurodevelopmental and neurodegenerative trajectories across the lifespan.
- The integration of a variational quantum circuit (VQC) module introduces a structured high-order nonlinear parameterization mechanism that may facilitate richer feature interactions and improve the flexibility of latent representation learning for complex age-related structural variations.

**Abstract:**

**Background/Objectives**: This study introduces Lightweight ScaleDense–Transformer (LST-Net), a design-driven framework for whole-lifespan brain age prediction, aiming to improve prediction accuracy while maintaining structural and biological plausibility. **Methods**: LST-Net utilizes a ScaleDense–Transformer architecture specifically engineered to mirror the dual-nature of brain aging: ScaleDense modules capture fine-grained local anatomical changes, while Transformers model global, network-level reorganizations. The framework was developed and evaluated using a modeling cohort of 22,271 subjects from 17 heterogeneous data sources, covering a 96-year lifespan. Additionally, an auxiliary quantum module (VQC) was incorporated within the latent bottleneck to facilitate nonlinear latent representation transformation and improve representation expressiveness. Performance was primarily assessed using Mean Absolute Error (MAE). **Results**: LST-Net achieved a competitive MAE of 2.71 years in brain age prediction, demonstrating stability across a wide lifespan (0–96 years) and varying data sources. Longitudinal assessments and test–retest analysis further confirmed the model’s high reproducibility, yielding an Intraclass Correlation Coefficient (ICC) of 0.994. **Conclusions**: The current findings establish LST-Net as a scalable tool for brain health monitoring and large-scale screenings.

## 1. Introduction

Brain aging is an intricate biological process characterized by progressive neuroanatomical alterations, including cortical thinning, ventricular enlargement, and white matter degradation [1,2]. Consequently, as a multifaceted biological proxy for these structural shifts, brain age—estimated from T1-weighted magnetic resonance imaging (MRI)—represents the manifested maturation or senescence level of the brain tissue [3,4,5]. Furthermore, by comparing this estimated brain age with the individual’s chronological age, the resulting “brain age gap” (or BrainAGE) quantifies deviation from a normative aging model [6,7]. A negative deviation suggests relatively preserved brain structure, whereas a positive deviation indicates an apparent age acceleration. Such acceleration has been associated with increased vulnerability to neurodegenerative processes [8,9] and may precede clinically observable abnormalities [10,11]. However, achieving high-precision brain age estimation remains a formidable challenge, primarily because aging-related signals are often subtle, spatially heterogeneous, and inherently non-linear [12,13]. Traditional predictive modeling, while effective in capturing macro-structural atrophy, frequently struggles to distinguish genuine senescent patterns from inter-individual morphological variability. From a methodological perspective, brain age estimation is not merely a conventional regression task; rather, it is an advanced representation learning problem. The accuracy of the final age prediction is fundamentally constrained by the model’s capacity to map high-dimensional, noisy voxel data into a discriminative and robust latent feature space [14]. Consequently, recent scholarly focus has shifted from simple volumetric statistics toward developing sophisticated architectures capable of extracting high-order non-linear representations. By refining the representational capacity of deep learning (DL) frameworks, it becomes possible to capture the complex, synergistic aging signatures distributed across the brain, ultimately leading to more stable and precise age estimations in diverse populations [13,15,16].

Guided by the evolving imperatives of representation learning, brain age estimation has progressed through several technological stages. Initially, traditional machine learning (ML) paradigms estimated brain age by mapping predefined neuroimaging features to chronological timelines. This is exemplified by Da Costa et al., who integrated shallow regressors with genetic hyperparameter tuning to attain a mean absolute error (MAE) of approximately 3.76 years during the PAC 2019 competition [17]. However, these approaches are limited by their reliance on manually engineered features, which often fail to capture high-dimensional, non-linear relationships in raw 3D MRI data. To overcome these limitations, DL has become the dominant paradigm, emphasizing automated hierarchical feature extraction. Within 3D-CNNs, the Simple Fully Convolutional Network (SFCN) [18] further enhances representational efficiency. By leveraging a compact kernel structure, SFCN and its variants like SFCN-reg effectively capture essential neuroanatomical atrophy patterns while minimizing computational redundancy [19]. Building upon these structural insights, the three-dimensional dual-stream fully convolutional residual network (ds-FCRN) [20] was proposed to further enhance localized representational depth. By synergizing dual-stream hierarchical architectures with residual learning, the ds-FCRN facilitates a specialized feature extraction process that maintains high-resolution spatial information while expanding the network’s capacity to decode multi-variate morphological shifts. ResNet architectures [21] address gradient degradation through residual connections, improving the training of deeper networks. Inception-based frameworks like DeepBrainNet [22] use multi-scale kernels to capture diverse morphological features, highlighting subtle structural patterns linked to cognitive functions. Despite these advances, CNNs are inherently limited by local receptive fields, restricting their ability to model long-range interactions among distant brain regions. To address this, Transformer-based architectures [23] exploit self-attention mechanisms to establish dynamic global receptive fields. For instance, the Medical Transformer [24] decomposes 3D scans into multi-view 2D sequences for efficient relational modeling, while Zhu et al. [25] combined 3D ResNet with a dual-branch Cross-Vision Transformer (ResNet-CrossViT) to extract multi-scale aging signatures. Nevertheless, a representational ceiling remains. Current hybrid models often rely on shallow fusion or successive permutation strategies, which struggle to fully refine compact high-level features and capture stable global relationships in a computationally efficient way. In particular, most frameworks perform global modeling at high-resolution feature maps, leading to heavy computation and difficulty in maintaining robustness under multi-center MRI variations.

These challenges indicate that effective brain age estimation depends not only on combining multiple modeling mechanisms, but also on how they are structurally organized to work together. Existing approaches typically integrate convolutional and attention-based modules, yet these components are often combined in loosely structured stacking or parallel designs without explicit functional roles, which may lead to computational redundancy or suboptimal representation efficiency. In particular, how to coordinate multi-scale local feature extraction, complex non-linear feature interactions, and efficient global integration within a unified framework remains underexplored, especially under multi-center heterogeneity and distribution shifts. To address this, we propose a tri-branch collaborative framework that decomposes the representation learning task into three functionally distinct stages: (1) Spatial Encoding, using a ScaleDense backbone to preserve multi-scale anatomical fidelity; (2) Semantic Refinement, employing a VQC-inspired structured nonlinear interaction module to perform parameter-efficient nonlinear transformation and implicit interaction modeling within the latent bottleneck representation space; and (3) Global Integration, utilizing a lightweight Transformer to harmonize long-range signatures.

The framework is designed to assign the aforementioned core tasks to dedicated components and to integrate them through a carefully structured data flow, aiming to yield complementary anatomical representations that span both fine-grained tissue structures and macroscopic brain topology. First, a ScaleDense 3D backbone is employed to extract hierarchical multi-scale anatomical features. By leveraging cross-scale dense connectivity, this module preserves fine-grained morphological information across multiple receptive fields, alleviates feature degradation in deep networks, and provides a high-fidelity multi-scale representation as a stable foundation for subsequent modeling. Second, within the compressed bottleneck space, we introduce a lightweight auxiliary interaction module. The motivation for this design lies in the observation that feature relationships in highly compressed semantic spaces may exhibit complex nonlinear dependencies, which can be challenging for traditional linear or shallow fully connected layers to effectively model. To enable more flexible interaction modeling, we adopt a quantum-inspired structured nonlinear parameterization module based on a variational quantum circuit (VQC) as one possible implementation. In this work, however, this module is employed as a parameter-efficient nonlinear transformation component for latent space regularization and representation refinement, rather than relying on its specific physical interpretation. Finally, for efficient global integration, a lightweight Transformer is constructed on the compressed semantic representations. By performing self-attention over a limited set of semantic tokens, this module captures long-range dependencies among distant brain regions at low computational cost, and integrates the outputs of the preceding stages into a coherent global representation. This design further contributes to improved stability and generalization under multi-center data conditions.

Overall, the proposed framework organizes the brain age estimation process into three coordinated stages: high-fidelity local encoding, bottleneck-level interaction enhancement, and efficient global integration. This structured design enables each component to focus on its primary function while operating collaboratively, thereby facilitating a more comprehensive and robust characterization of multi-scale brain aging patterns.

## 2. Materials and Methods

### 2.1. Datasets

We compiled a large, lifespan-spanning neuroimaging database by including only datasets with T1-weighted MRI and complete demographic information (age and sex) for healthy participants. For any constituent repositories originally collected for clinical or disease-specific studies (such as those investigating neurodegenerative, neuropsychiatric, or neurodevelopmental conditions), we strictly extracted only the clinically unaffected healthy controls, while explicitly excluding any individuals with active pathological diagnoses. A total of 27 datasets met these criteria. To improve model generalizability and robustness, variance in technical acquisition profiles—encompassing diverse manufacturer hardware, spatial resolutions, and sequence designs—was intentionally maintained.

All data were categorized according to their role in model development, allowing readers to readily understand each dataset’s intended function. When a dataset served multiple purposes—for example, both within the modeling cohort and for longitudinal or repeat-scan analyses—participants involved in longitudinal or repeat-scan measurements were prioritized for assignment to the corresponding evaluation subsets, and any overlapping samples were subsequently removed from the modeling cohort. This strategy minimizes the risk of data leakage and supports unbiased performance assessment. The MRI data were ultimately organized into two primary cohorts: The modeling cohort is used for model training and optimization, was further partitioned into a training set (70%) to update network weights, a validation set (15%) dedicated to hyperparameter adjustment and executing early stopping criteria, and an internal evaluation set (15%) earmarked for preliminary performance assessment within the modeling cohort. Evaluation cohort is reserved for independent testing of model generalization and reliability, further subdivided into three task-specific sets: Cross-site set–MRI scans from centers not included in the modeling cohort, used to evaluate generalization across imaging sites. Longitudinal set leverages sequential MRI sessions from the same subject cohort at distinct chronological intervals, serving to verify the long-term consistency of our estimations. Repeat-scan set–repeated scans of the same participants within a short interval, used to measure reliability and repeatability. Every MRI volume was subjected to strict visual auditing to exclude scans exhibiting noticeable movement degradation or anomalous signal variations. The final cohort included healthy participants aged 0–96 years, covering the full human lifespan. The modeling cohort supported model training and parameter optimization, whereas the evaluation cohort provided an independent, unbiased assessment of model performance. To completely untangle the multi-use subsets, we guarantee that the subject identities are strictly mutually exclusive across the training, validation, internal evaluation, cross-site, longitudinal, and repeat-scan sets. No individual or associated longitudinal/retest session appears in more than one partition. Detailed characteristics and specific information of all datasets are presented in Supplementary Materials Table S1, and the age distribution of each dataset is illustrated in Supplementary Materials Figure S1, with subfigures (a) to (e) corresponding to the sex-stratified age distribution of the modeling cohort (*n* = 22,271), age distribution of the training set of the modeling cohort (*n* = 15,582), age distribution of participants in the validation set of the modeling cohort (*n* = 3341), age distribution of the internal evaluation set (*n* = 3348), and age distribution of the cross-site set (*n* = 1422), respectively.

### 2.2. Data Preprocessing

T1-weighted MRI preprocessing was carried out in MATLAB R2019a using the CAT12 toolbox (Computational Anatomy Toolbox for SPM, version 12.8; https://neuro-jena.github.io/cat/, accessed on 20 February 2024), which operates within the SPM12 (Statistical Parametric Mapping, version v7771; https://www.fil.ion.ucl.ac.uk/spm/software/, accessed on 20 February 2024) environment. All preprocessing and structural normalization procedures were conducted independently at the set level strictly after the dataset partitioning stage. This ensures that the different datasets remained mathematically isolated throughout the entire pipeline. The processing stream leveraged the standard CAT12 architecture to execute both comprehensive tissue partitioning and uniform spatial normalization. Specifically, raw images underwent automated bias-field correction to mitigate radio-frequency spatial inhomogeneities and explicit intensity normalization via the built-in AMAP (Adaptive Maximum A Posterior) approach to standardize the tissue intensity metrics across multi-center data. Brain tissue was isolated through an automated skull-stripping graph-cut algorithm. Subsequently, high-dimensional DARTEL non-linear registration was employed to align individual native anatomical scans into the standard MNI151 template (IXI555 space). To preserve fine-grained localized structural boundaries and high-frequency anatomical features for the downstream deep learning kernels, no spatial smoothing was applied to the tissue segments.

To guarantee high data fidelity, a rigorous two-step quality control (QC) protocol was executed. First, the CAT12 image quality score (IQR) calculated based on noise and inhomogeneity metrics, was applied for objective screening. Data samples failing to reach a threshold of 75 were systematically discarded. For the remaining high-scoring volumes, we implemented a manual visual inspection to identify and eliminate subtle regional segmentation faults or severe motion-induced blurring. This two-stage QC process effectively preserved the structural integrity of tissue segmentation and spatial warping.

After preprocessing, gray matter density maps (GMDM) were generated at an isotropic spatial resolution of 1 × 1 × 1 mm^3^ (matrix dimensions: 169 × 205 × 169 pixels per volume). To control for morphological variations and total brain volume variance across individuals, a modulation step was integrated, producing volume-corrected structural maps optimized for downstream tasks. Furthermore, to mitigate computational overhead and stabilize network convergence, these modulated maps underwent spatial dimension scaling down to an 84 × 102 × 84 grid (isotropic 2 × 2 × 2 mm^3^ voxels) via trilinear interpolation. This reduction compressed the data footprint while maintaining core anatomical topography. Sex, scanner variations, and total intracranial volume (TIV) were retained as intrinsic features within the dataset; no pre-regressed site harmonization or covariate corrections were applied before network input, thereby empowering the network to adaptively capture site-blind aging signatures while robustly handling multi-site heterogeneity.

### 2.3. Performance Metrics

To systematically validate the model across various practical conditions, three analytical dimensions—namely predictive precision, temporal consistency, and test–retest reproducibility—were quantified using distinct, task-specific metrics. These evaluations were implemented across four functionally segregated subsets derived from our dataset partitioning: an internal evaluation set, a cross-site dataset, a longitudinal set, and a repeat-scan set.

#### 2.3.1. Accuracy and Robustness

For both the internal evaluation set and the independent unseen dataset, model performance was evaluated in terms of predictive accuracy and robustness using multiple quantitative metrics. Prediction accuracy was primarily assessed by the MAE.

(1) $$MAE=\frac{1}{N}\sum_{i=1}^{N}\left|{\hat{y}}_{i}-y_{i}\right|$$

where $y_{i}$ denotes the ground-truth age of the i−th subject, ${\hat{y}}_{i}$ denotes the corresponding predicted age, and N is the total number of subjects. A smaller MAE reflects better predictive accuracy.

To further characterize model performance, we computed the mean error (ME) to quantify systematic bias—specifically, whether the model exhibits a consistent tendency to overpredict or underpredict age:

(2) $$ME=\frac{1}{N}\sum_{i=1}^{N}\left({\hat{y}}_{i}-y_{i}\right)$$

Here, an ME value near zero indicates minimal systematic bias, while positive and negative values reflect a general tendency toward age overestimation and underestimation, respectively.

To assess performance stability across different age ranges, we further employed the maximal MAE (mMAE). This metric first divides subjects into age bins based on chronological age, computes the MAE within each bin, and then takes the maximum value across all bins. Formally, given *K* age bins, and the k-th bin is $\left[b_{k},b_{k+1}\right)$. Let $I_{k}=\{i|y_{i}\in [b_{k},b_{k+1})\}$ denote the set of indices belonging to the k-th bin, and let $N_{k}=\left|I_{k}\right|$ represent the number of samples in that bin. The MAE for the k-th bin is expressed as:

(3) $${MAE}_{k}=\frac{1}{N_{k}}\sum_{i\in I_{k}}\left|{\hat{y}}_{i}-y_{i}\right|$$

The final mMAE is consequently defined as:

(4) $$mMAE=\underset{k=1,\ldots ,K}{max}{MAE}_{k}$$

Here, *K* is the number of age bins; $b_{k}$ and $b_{k+1}$ define the lower and upper boundaries of the k-th bin, respectively; $I_{k}$ represents the set of sample indices within that interval; $N_{k}$ is the number of subjects in the bin; and ${MAE}_{k}$ corresponds to the mean absolute error computed for that specific bin. The mMAE thus captures the largest prediction error observed across age strata. A close correspondence between mMAE and the overall MAE indicates that the model maintains consistent performance across different age groups, reflecting good robustness and balanced generalization. In this study, the test-set chronological ages spanned from 0 to 96 years. Subjects were stratified into 10-year age bins, with a separate final bin defined for the remaining upper range that did not constitute a full interval. All bins were defined using a left-inclusive, right-exclusive convention. We computed MAE independently within each age interval, and initially defined mMAE as the maximum MAE across all ten bins. However, only *n* = 2 subjects fell into the [90, 96) interval, making this bin highly sensitive to outliers and potentially inflating the mMAE. To avoid distortion from such an extremely small sample size and to provide a more reliable estimate of worst-case performance, this bin was excluded from the final calculation. The reported mMAE therefore corresponds to the highest MAE observed across the remaining age intervals. This exclusion was performed solely to avoid statistically unstable and high-variance error estimation due to the extremely limited sample size in this interval, rather than to artificially improve apparent model robustness. All subjects in the [90–96) age bin were still included in model training, validation, and the global performance metrics.

In addition, we used the Pearson correlation coefficient (*r*) to assess the strength of the linear relationship between predicted and true ages, and the coefficient of determination (*R*^2^) was employed to measure how much variance in the true ages is accounted for by the model outputs:

(5) $$r=\frac{\sum_{i=1}^{N}\left(y_{i}-\bar{y}\right)\left({\hat{y}}_{i}-\bar{\hat{y}}\right)}{\sqrt{\sum_{i=1}^{N}{\left(y_{i}-\bar{y}\right)}^{2}}\sqrt{\sum_{i=1}^{N}{\left({\hat{y}}_{i}-\bar{\hat{y}}\right)}^{2}}}$$

(6) $$R^{2}=1-\frac{\sum_{i=1}^{N}{\left(y_{i}-{\hat{y}}_{i}\right)}^{2}}{\sum_{i=1}^{N}{\left(y_{i}-\bar{y}\right)}^{2}}$$

Here, $\bar{y}$ denotes the mean of the true ages and $\bar{\hat{y}}$ denotes the mean of the predicted ages. In general, values of |r| approaching 1 indicate a stronger linear association, while higher *R*^2^ values reflect better goodness of fit and greater explanatory power of the model in capturing the variance of chronological age.

#### 2.3.2. Consistency

For the longitudinal dataset, we evaluated model consistency using a set of dedicated metrics. In healthy individuals, the change in predicted brain age between follow-up scans should ideally correspond to the actual time elapsed. To assess this, for the i-th subject with two scans—baseline (B) and follow-up (F)—we quantified the deviation between the predicted age difference and the true chronological interval. The definitions are as follows:

True change: $\Delta y_{i}=y_{i2}-y_{i1}$

Predicted change: $\Delta {\hat{y}}_{i}={\hat{y}}_{i2}-{\hat{y}}_{i1}$

Change error: $\delta_{i}=\Delta {\hat{y}}_{i}-\Delta y_{i}$

We further defined the Mean Difference Error (MdE), Mean Absolute Difference Error (MAdE), and Maximal Mean Absolute Difference Error (mMAdE) as follows:

(7) $$MdE=\frac{1}{N}\sum_{i=1}^{N}\left[\left({\hat{y}}_{i2}-{\hat{y}}_{i1}\right)-\left(y_{i2}-y_{i1}\right)\right]=\frac{1}{N}\sum_{i=1}^{N}\delta_{i}$$

(8) $$MAdE=\frac{1}{N}\sum_{i=1}^{N}\left|\left({\hat{y}}_{i2}-{\hat{y}}_{i1}\right)-\left(y_{i2}-y_{i1}\right)\right|=\frac{1}{N}\sum_{i=1}^{N}\left|\delta_{i}\right|$$

(9) $${MAdE}_{k}=\frac{1}{N_{k}}\sum_{i\in I_{k}}\left|\delta_{i}\right|$$

(10) $$mMAdE=\underset{k=1,\ldots ,K}{max}MAdE_{k}$$

where $y_{i1}$ and $y_{i2}$ denote the true chronological ages of subject *i* at B and F, respectively; ${\hat{y}}_{i1}$ and ${\hat{y}}_{i2}$ denote the corresponding predicted brain ages; $\Delta y_{i}$ represents the true F interval; $\Delta {\hat{y}}_{i}$ represents the predicted F interval; $\delta_{i}$ is the consistency error. Physically, MdE values approaching zero suggest reduced systematic bias, while lower MAdE and mMAdE values reflect improved longitudinal consistency and greater reliability in capturing individual aging trajectories.

#### 2.3.3. Reproducibility

For the test–retest dataset, reproducibility was assessed using a set of dedicated metrics. A model is regarded as highly reproducible when it yields stable predictions for the same individual across repeated scans. For each subject *i*, let ${\hat{y}}_{i1}$ and ${\hat{y}}_{i2}$ denote the predicted brain ages from the first and second scans, respectively. We first compute variability by calculating the standard deviation between the two predicted values

(11) $$\sigma_{i}=SD\left({\hat{y}}_{i1},{\hat{y}}_{i2}\right)$$

We computed the mean of this measure across all subjects who had paired test–retest scans:

(12) $$\sigma \left({\hat{y}}_{scan}^{\prime }\right)=\frac{1}{M}\sum_{i=1}^{M}\sigma_{i}$$

where *M* is the number of subjects with paired scans. A smaller $\sigma \left({\hat{y}}_{scan}^{\prime }\right)$ indicates minimal prediction fluctuations for the same subject, reflecting higher predictive stability.

In addition, we use the mean difference *μ*(*d*) to assess whether there is a systematic shift between the two repeated measurements:

(13) $$\mu \left(d\right)=\frac{1}{N}\sum_{i=1}^{N}d_{i=}\frac{1}{N}\sum_{i=1}^{N}{\hat{y}}_{i1}-{\hat{y}}_{i2}$$

In theory, a *μ*(*d*) *value* near zero implies no systematic bias. A positive *μ*(*d*) (*μ*(*d*) > 0) means the first-scan predictions tend to be higher than those from the second scan on average, whereas a negative *μ*(*d*) (*μ*(*d*) < 0) indicates reverse pattern.

To characterize the dispersion of test–retest differences, we compute the standard deviation of differences *σ*(*d*); smaller values indicate greater stability:

(14) $$\sigma \left(d\right)=SD\left(d_{1},d_{2},\ldots ,d_{N}\right)$$

Finally, the Intraclass Correlation Coefficient (ICC) is employed to quantify both agreement and reliability under repeated measurements. We adopted ICC(3,1) (two-way mixed-effects model, consistency definition, single measurement) to evaluate test–retest reproducibility, defined as:

(15) $$ICC\left(3,1\right)=\frac{MS_{subject}-MS_{error}}{MS_{subject}+\left(k-1\right)MS_{error}}$$

where $MS_{subject}$ is the mean square between subjects, $MS_{error}$ is the residual mean square, and *k* is the number of repeated measurements (here *k* = 2). Note that *ICC*(3,1) under the consistency definition primarily evaluates whether the relative ranking of subjects’ predictions remains stable across repeated scans, rather than requiring strict numerical equality between the two predicted values. To compensate for this conceptual boundary, our evaluation framework concurrently utilizes the aforementioned $\sigma \left({\hat{y}}_{scan}^{\prime }\right)$, μ(d), σ(d) to rigorously monitor absolute value fluctuations and restrict potential systematic drifts between sessions. Consequently, this multi-metric tracking ensures a comprehensive validation of both absolute numerical alignment and relative rank-order stability.

### 2.4. Model Architecture

In this study, we propose a hierarchical hybrid framework, termed Lightweight ScaleDense–Transformer (LST-Net). The core design philosophy of LST-Net lies in a functionally partitioned collaborative system that synergizes the respective strengths of CNNs, quantum-inspired compact nonlinear parameterization modules, and Transformers at distinct representational levels.

**Figure 1:**
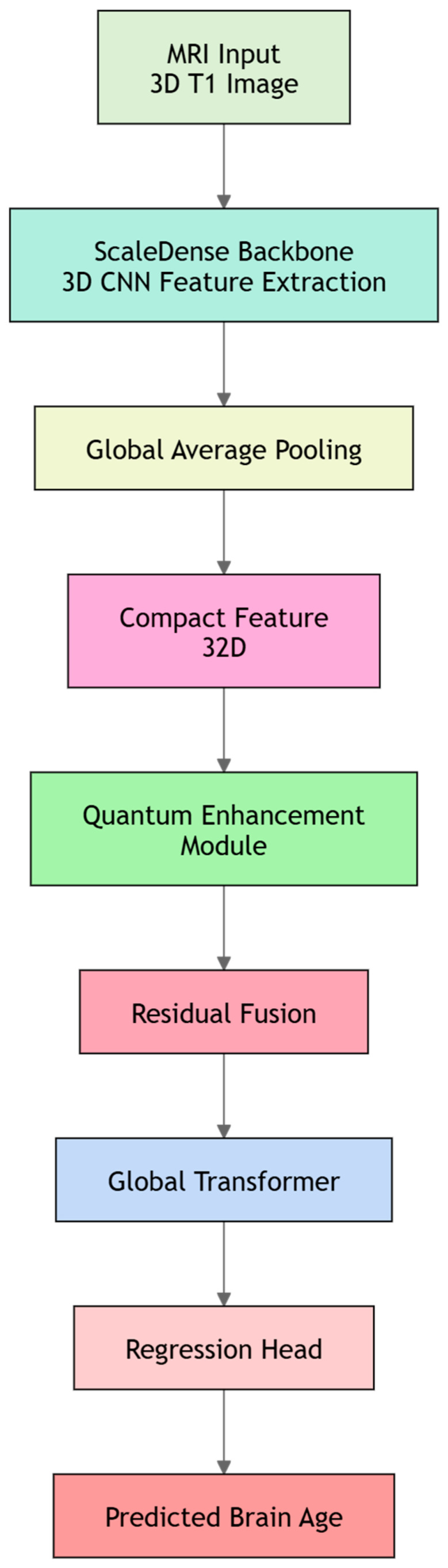
Architectural Blueprint of the LST-Net Framework.

As illustrated in Figure 1, the logical architecture follows a sequential pipeline: spatial encoding, semantic refinement, and global integration. The framework comprises three functionally dedicated branches: Spatial Encoding Branch: A 3D ScaleDense backbone is employed to extract hierarchical, multi-scale anatomical features directly from preprocessed GMDM voxels. By leveraging its local inductive bias, this module establishes a robust spatial representation foundation. Semantic Refinement Branch: A VQC-based structured nonlinear interaction module is introduced within the compressed bottleneck space strictly as a compact non-linear latent transformation module. Utilizing parameter-efficient and bounded nonlinear mapping structure, this module provides effective implicit regularization in the latent representation space, facilitating feature reorganization and refinement of the classical representations. Global Integration Branch: Operating on the tokenized semantic space, a lightweight Transformer module captures long-range contextual associations among distant brain regions. It harmonizes the multi-scale signatures to produce the final brain age estimation. By constraining complex global modeling to a low-dimensional semantic manifold, this tri-branch design ensures high expressivity while maintaining computational efficiency, making it well-suited for whole-lifespan brain age prediction across diverse populations. To provide a clear structural blueprint, the exact structural specifications, localized tensor mapping transformations, and block-wise parameter allocations across all LST-Net branches are systematically cataloged and detailed in Supplementary Materials Table S2.

#### 2.4.1. Hierarchical Spatial Encoding via ScaleDense Backbone

To effectively capture the intricate and multifaceted structural shifts associated with brain aging, the LST-Net employs a specialized ScaleDense backbone as its primary feature extraction engine. The architecture is rooted in the DenseNet paradigm [26], which emphasizes maximal information flow through continuous feature integration. Unlike traditional linear CNNs that operate like a “relay race”—where each layer receives information only from its immediate predecessor and risk “washing away” original structural cues—the ScaleDense backbone functions as a collective memory. By establishing direct pathways from every preceding layer to all subsequent stages, the network ensures that fine-grained morphological details, such as localized cortical thinning, are explicitly “carried forward” and synthesized with high-level semantic patterns.

The encoding process begins with an initial 3D convolutional layer utilizing a wide 7 × 7 × 7 receptive field with a stride of 1, padding of same, and dilation of 2. This configuration, followed by an Exponential Linear Unit (ELU) activation function, allows the model to immediately sense broad, low-frequency structural patterns and spatial organizational relationships across the brain volume without sacrificing resolution. Following this initial encoding, the data flows through five sequential dense stages (*nb_block* = 5).

The core mathematical principle of these stages is recursive concatenation. In each stage, the network generates a set of new features with twice the width of the input channels and appends them to the existing reservoir of historical features. To ensure that this “snowballing” effect does not lead to a computational explosion in 3D space, we implement a parsimonious growth strategy. By initiating the network with a narrow width of nb_filter = 8 and tripling the total channel cardinality at each stage, the feature depth follows a geometric progression from 8 to 1944 (8→24→72→216→648→1944). Crucially, to maintain spatial consistency during fusion, both the new feature path and the historical reuse path undergo a 2 × 2 × 2 Max-pooling operation before being concatenated. This ensures that the model can simultaneously leverage “fine-grained, early-stage” structural cues from shallow layers and “abstract” aging signatures from deeper ones at a synchronized spatial resolution.

An innovation within each dense stage is the integration of Asymmetric Convolutional (AC) Blocks. Recognizing that brain structures—such as the convoluted folds of the sulci, the ridges of the gyri, and the orientation of white matter tracts—are inherently anisotropic, we replace standard isotropic kernels with a four-branch parallel architecture. In this framework, a standard 3 × 3 × 3 kernel is synergized with three orthogonal filters (1 × 1 × 3, 3 × 1 × 1, and 1 × 3 × 1) aligned with the primary anatomical axes. To ensure stable optimization, each branch is equipped with Batch Normalization (BN) before fusion. These directional branches act as “anatomical edge-finders,” specifically tuned to capture morphological shifts that extend along specific spatial orientations. By fusing these four branches through a process of element-wise summation, the AC-block achieves a multi-directional field of view without inflating the network’s parameter footprint. Crucially, the first AC-block in each stage also serves as the primary dimensionality expander, projecting the input channels into a wider representation. Within a single dense stage, two such AC-Blocks are stacked consecutively, each followed by BN and ELU layers, to provide a deep, non-linear distillation of localized anisotropic features while doubling the feature density of the newly generated path.

Within each dense stage, the newly extracted features undergo dynamic recalibration via a Squeeze-and-Excitation (SE) block. This mechanism functions as a “semantic filter” by performing a global descriptor aggregation through 3D adaptive average pooling. It calculates the relative importance of each feature channel and adaptively prioritizes those most discriminative for age estimation while suppressing redundant morphological noise. Specifically, with a reduction ratio of 16, the SE-block models the intricate interdependencies among channels, refining the high-level semantics of the current stage before they are pooled and integrated into the historical feature stream. Critically, by recalibrating only the incremental features and bypassing the identity path, the model preserves the integrity of the historical feature reservoir. This targeted refinement maintains the cumulative stability of the dense growth mechanism while ensuring that only the most age-informative signatures are emphasized before they are pooled and integrated into the broader feature stream.

**Figure 2:**
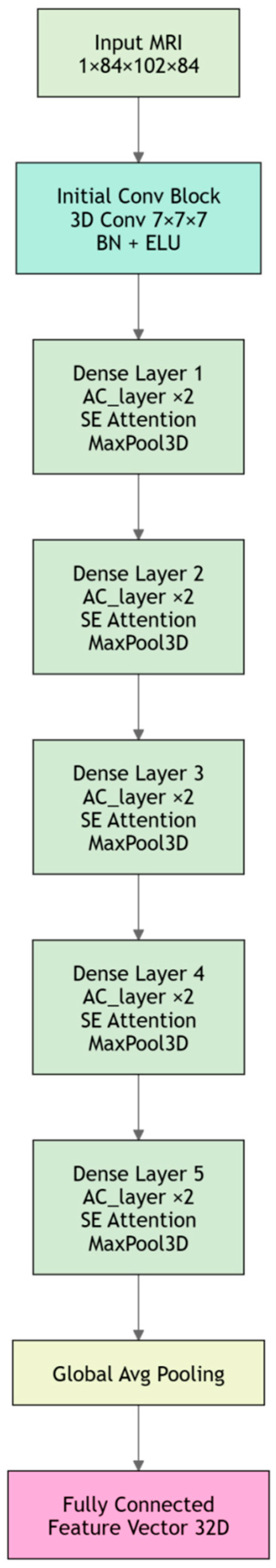
Modular composition and data flow visualizer of the ScaleDense backbone.

At the terminus of the ScaleDense backbone, the accumulated multi-scale feature maps represent a highly abstracted summary of the brain’s status. To bridge the gap between volumetric spatial encoding and compact semantic modeling, the architecture employs Global Adaptive Average Pooling (GAP). This operation collapses the spatial dimensions into a unified feature vector, which is then projected through a final linear transformation into a 32-dimensional latent bottleneck. This compact vector serves as the “Global Representation,” encapsulating the most salient morphological signatures extracted by the backbone. By distilling high-dimensional voxel data into this discriminative and lightweight manifold, the backbone provides a stable foundation for the subsequent quantum-inspired refinement and global Transformer harmonization stages. A comprehensive schematic of the ScaleDense backbone, is presented in Figure 2.

#### 2.4.2. Latent Interaction Refinement Using VQC-Based Bottleneck

Following the spatial encoding process, the LST-Net introduces a specialized refinement stage designed to model complex interdependencies within the compact latent space. We term this module the VQC-based Bottleneck, which leverages the structured mathematical framework of VQC as a sophisticated non-linear mapping unit. The core rationale is to structured parameterized nonlinear transformation module to capture complex feature interactions beyond standard element-wise activations. The model refines latent representations through sequential differentiable mapping operations within a compact latent space, leading to more discriminative features before global integration.

The refinement process begins by projecting the 32-dimensional classical vector into an 8-dimensional (n_qubits = 8) workspace via a linear layer. To ensure compatibility with the variational gate parameters, the output of the projection layer is first constrained by a tanh activation to the $[−1, 1]$ interval and subsequently scaled by pi, effectively mapping them into the angular interval of [−pi, pi]. These values serve as the input for Angle Embedding, specifically utilizing Y-axis rotations (*Ry*) to encode the signals into the 8 virtual qubits. This “32-8-32” bottleneck architecture is a deliberate design choice to control the scale of the refinement branch, ensuring that the VQC acts as a highly regularized, parameter-efficient feature refiner without overwhelming the model’s computational footprint.

The heart of the bottleneck is a variational circuit consisting of two layers (q_layers = 2). Within each layer, every dimension undergoes a parameterized transformation via a three-variable rotation gate, Rot(phi, theta, omega). To facilitate deep interaction between different feature dimensions, we implement a circular entanglement topology using Controlled-NOT gates. In this configuration, each qubit is sequentially coupled with its neighbor in a closed loop (q→q + 1), with the last linked to the first. This topology functions as a structured feature mixer, allowing the model to capture multi-channel dependencies through a fixed geometric hierarchy. This arrangement is chosen over all-to-all connectivity to minimize circuit depth and mitigate the risk of training instabilities, such as vanishing gradients, while maintaining sufficient cross-dimensional information propagation.

**Figure 3:**
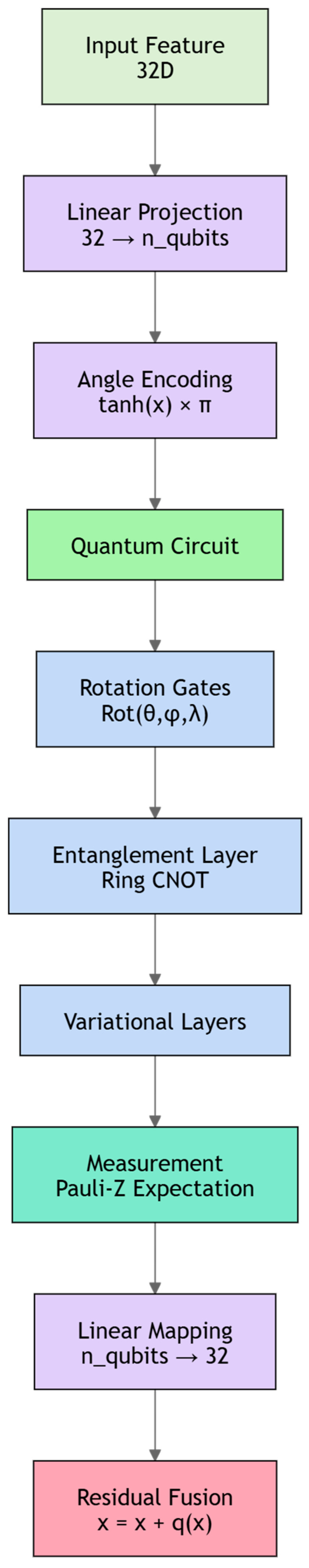
Detailed configuration of the VQC-based Bottleneck for latent interaction refinement.

Upon completion of the circuit, the expectation values of the Pauli-Z operator are measured for each dimension, yielding a refined 8-dimensional vector. This output is projected back to 32 dimensions and integrated with the original representation via an additive skip connection. This ‘by-pass’ architecture ensures that the VQC-based branch functions as a supplementary refiner rather than a disruptive replacement. Crucially, this design prioritizes the stable representations of the classical backbone; during the initial stages of training, the predictive flow is primarily dominated by backbone features, which contributes to stable optimization and disrupting early-stage representation learning dynamics. This design treats the VQC component as a parameter-efficient, structured nonlinear transformation module for enhancing feature expressiveness within the LST-Net framework. The detailed schematic of this VQC-based Bottleneck is depicted in Figure 3.

#### 2.4.3. Global Feature Integration with Lightweight Transformers

Following the integration of spatial features and quantum-inspired refinements, the LST-Net introduces a Global Transformer module at the terminus of the architecture. This module implements a Post-Semantic Tokenization strategy. By restricting the attention mechanism to the compact 32-dimensional latent space, the Transformer serves as a lightweight relational aggregator rather than a primary feature extractor, specifically designed to model non-local dependencies between distinct semantic subspaces.

The harmonization process begins by restructuring the 32-dimensional fused feature vector into a sequence of tokens. Defined by a sequence length of *trans_seq_len* = 8, the latent vector is uniformly partitioned into eight discrete “semantic slots,” each possessing a dimensionality of $d_{model}$ = 4. Crucially, this tokenization is achieved through a deterministic reshaping operation rather than learnable linear projections. This design ensures that the Transformer’s capacity is dedicated entirely to modeling the interactions between fixed semantic regions, avoiding the introduction of projection uncertainties at the bottleneck stage. To distinguish these slots, a learnable Position Embedding (pos_embed) is added to the sequence. Although these tokens do not represent explicit 3D spatial coordinates, the embedding provides a unique identity bias for each slot, preventing the self-attention mechanism from treating the features as a permutation-invariant set. This allows the model to preserve the underlying structural hierarchy inherent in the backbone’s output while re-evaluating the global context.

The internal architecture consists of *trans_layers* = 2 Transformer encoder layers, utilizing a Pre-Norm configuration (norm_first = True) to enhance training stability. Each layer integrates a multi-head self-attention (MHSA) sub-module with trans_heads = 2 and a feed-forward network (FFN) utilizing GELU activations. Within this framework, each attention head operates on a 2-dimensional subspace, performing a lightweight re-calibration of the semantic slots. This parsimonious configuration is a deliberate engineering choice: rather than learning high-capacity visual representations, the MHSA focuses on answering which low-dimensional semantic components are most relevant to the final age estimation task.

**Figure 4:**
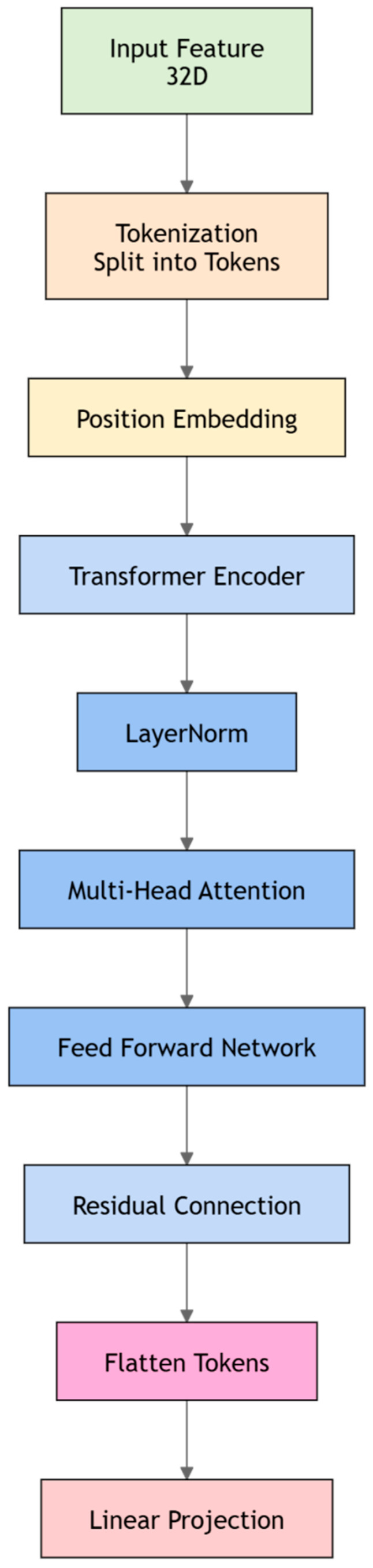
Detailed architecture of the Lightweight Transformer.

The output of the Transformer sequence is flattened and projected back to the original 32-dimensional space via a linear transformation. This refined signal is then integrated with the module’s input through an additive residual connection, followed by a final LayerNorm for stabilization. This “Global Correction” framework ensures that the Transformer functions as a relational fine-tuner, augmenting the established representations of the ScaleDense and quantum branches without disrupting their stable structural cues. The final age prediction is generated by a streamlined Regression Head. The 32-dimensional harmonized vector is first compressed into a 16-dimensional manifold to synthesize the global semantics, followed by a linear mapping to a single scalar. A ReLU activation is applied to the final output to enforce a non-negative constraint, aligning the model’s predictions with the biological reality of chronological age. The detailed architectural configurations of the Transformer module a are illustrated in Figure 4.

#### 2.4.4. Implementation Details

The LST-Net framework was implemented using PyTorch 2.0.1 and PennyLane 0.32.0, executed on a workstation equipped with an NVIDIA RTX 3090 (24 GB) GPU and Intel Xeon CPU (CUDA 11.8/cuDNN 8.7). The quantum-inspired refinement module utilized the default.qubit simulator backend and was integrated into the PyTorch computational graph via the qml.qnn. TorchLayer interface. This setup enabled joint end-to-end optimization of both classical and variational parameters without freezing the backbone. To further enhance parameter stability, an Exponential Moving Average (EMA) mechanism with a 0.999 decay was incorporated throughout the process. Due to the massive dataset scale, the full end-to-end training process requires approximately 3 weeks of continuous execution to achieve global convergence.

The final configuration employed the AdamW optimizer with a batch size of 8 and a weight decay of 0.001. We utilized a OneCycleLR schedule with a peak learning rate of 3 × 10^−4^, applying a cosine annealing strategy for dynamic adjustment. The joint objective function integrated MAE as the primary regression loss and an auxiliary ranking loss (weight 0.2). Notably, the ranking loss was introduced via a progressive strategy—remaining inactive for the first 8 epochs and then linearly scaling up to its full weight over the next 30 epochs to prevent interference during early training. A soft-cap mechanism (threshold 20.0) was also applied to suppress disruptive loss spikes.

Training was limited to 100 epochs with an early stopping patience of 15. To avoid being misled by initial training fluctuations, the early stopping monitor was only activated after 30 epochs, ensuring the selection of the most robust model based on validation performance.

## 3. Results

To rigorously quantify the impact of each architectural innovation in LST-Net, we performed a series of ablation experiments. This process involved comparing the complete framework against three distinct baseline configurations designed to isolate specific functional modules:

ScaleDense-only (Baseline 1): This configuration utilizes only the 3D ScaleDense convolutional backbone and the regression head, completely omitting the quantum-inspired refinement and Transformer layers. It serves to establish a performance benchmark for hierarchical spatial feature extraction, evaluating how effectively localized anatomical patterns alone can support brain age estimation.

Transformer-only (Baseline 2): To assess the impact of global contextual modeling in the absence of convolutional inductive biases, this baseline employs a standard 3D Transformer encoder for whole-brain neuroimaging. The input MRI volume is embedded into visual tokens via a linear patch projection layer, followed by a multi-layer, multi-head Transformer encoder to capture long-range structural dependencies. Specifically, the input volume is partitioned into regular 4 × 4 × 4 non-overlapping patches and mapped via a linear projection layer into a 32-dimensional token space. These tokens are processed through a 4-layer, 2-head 3D Transformer encoder with a FFN dimension of 64 to capture long-range structural dependencies. The resulting token representations are aggregated and passed to a regression head for brain age prediction. This design serves as a Transformer-based reference architecture for evaluating the capability of pure self-attention mechanisms in modeling age-related structural patterns.

ScaleDense–Transformer (Baseline 3): This model integrates the convolutional features from ScaleDense with the global semantic tokens from the Transformer module through a classical cascaded architecture, completely excluding the VQC-based bottleneck. Specifically, the 32-dimensional spatial feature vector derived from the backbone is compressed and then restored through a classical 32 to 8 to 32 multi-layer bottleneck projection, integrated with intermediate ReLU. Instead of passing through a virtual quantum circuit, these classically rectified non-linear spatial features are subsequently fed directly as token slot inputs into the Transformer encoder layers for global attention modeling. By serving as a classical-only hybrid serial reference with an identical bottleneck topology, this baseline allows for a quantitative evaluation of the marginal performance improvements specifically driven by the integration of the quantum-inspired refinement branch.

To maintain experimental rigor, all ablated versions were optimized using a unified training pipeline, data partitioning scheme, and preprocessing workflow identical to those employed for the full LST-Net framework.

### 3.1. Performance Evaluation on the Internal Evaluation Set

As illustrated in Table 1, the proposed LST-Net demonstrated improved quantitative results over all considered baseline configurations. It achieved an MAE of 2.71 years, a Pearson’s r of 0.98, and an R^2^ of 0.97. In comparison, the individual backbones—Scale Dense (MAE = 4.33 years) and Transformer (MAE = 5.19 years)—showed higher prediction errors and more pronounced systematic underestimation, as indicated by their negative ME values. Notably, the integration of the quantum-inspired component was associated with enhanced stability across different age intervals. The mMAE dropped from 15.98 years in the ScaleDense–Transformer baseline to 5.77 years in LST-Net, representing a quantitative reduction in the maximum error across age bins. These quantitative differences suggest that while traditional cascaded pipeline offers a baseline for hybrid modeling, the VQC-based bottleneck facilitates sophisticated feature recalibration, acting as a high-dimensional non-linear refiner that enhances the discriminative power of the bottleneck representation. Furthermore, the near-zero ME (0.13 years) achieved by LST-Net implies a highly calibrated prediction trajectory with minimal directional bias, reinforcing the framework’s reliability for consistent brain age estimation across the entire internal evaluation set.

**Figure 5:**
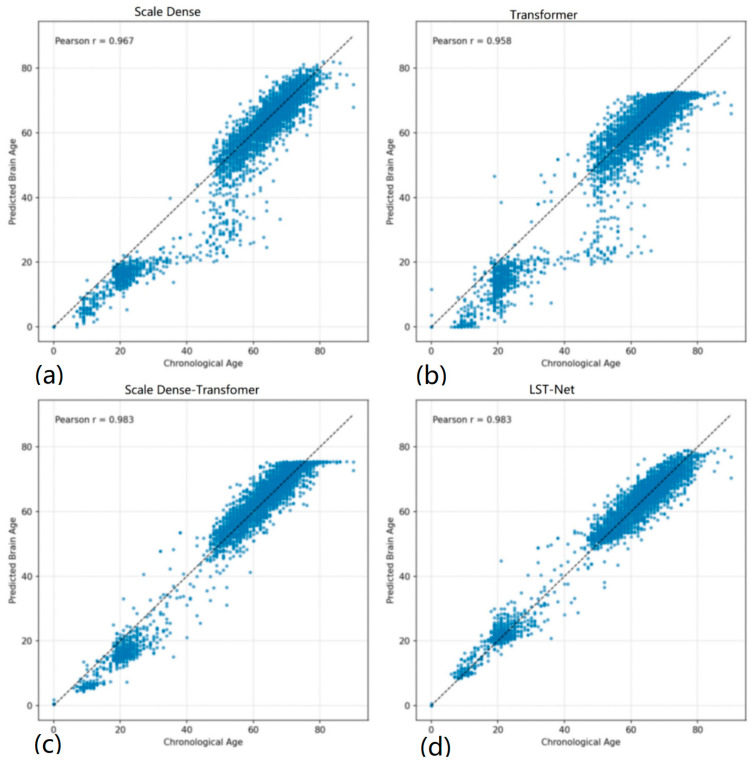
Predicted vs. chronological age scatter plots for the internal evaluation set. > Panels show results for (**a**) ScaleDense, (**b**) Transformer, (**c**) ScaleDense–Transformer, and (**d**) LST-Net. The dashed reference line denotes the ideal identity mapping. These visualizations highlight the comparative dispersion and systematic bias of each model relative to the true chronological age.

The scatter plots in Figure 5 further corroborate the quantitative results. While all models follow the general aging trend, the baseline ScaleDense and Transformer architectures exhibit prominent ‘prediction plateaus’ and increased dispersion, particularly at the chronological extremes. Specifically, these models show a marked tendency to underestimate the age of elderly subjects and struggle with the rapid structural transitions in the pediatric cohort. In contrast, LST-Net’s predictions are more tightly clustered around the identity line, forming a significantly narrower scatter band across the entire lifespan. This linear consistency suggests that the integration of the VQC-based refinement effectively rectifies regional biases and enhances the model’s sensitivity to subtle, non-linear morphological nuances that classical cascaded transformations fails to resolve, especially during the stable adulthood ‘plateau’ and accelerated senescent atrophy.

To evaluate the model’s sensitivity to diverse neuroanatomical transitions, we partitioned the internal evaluation set into three biologically distinct phases: neurodevelopment (youth), structural maturity (adulthood), and senescence (elderly).

Developmental Phase (Ages 0–18): This period is characterized by rapid structural remodeling. As shown in Table 2, LST-Net achieved an MAE of 1.82 years and an R^2^ of 0.62. In contrast, the Transformer-only baseline exhibited limited predictive capacity in this pediatric cohort, characterized by a higher MAE (7.28 years) and a negative R^2^ (−3.45). These results indicate that the quantum-inspired module, utilizing alternative mathematical representations in the latent space, provides a useful complement to classical feature extraction for modeling non-linear maturation trajectories.

Stable Adulthood (Ages 18–60): For the adult cohort (Table 3), LST-Net maintained consistent performance with an MAE of 2.70 years and an R^2^ of 0.95. While the standalone classical backbones showed persistent systematic underestimation (ME < −3.9 years), LST-Net exhibited a more balanced error distribution (ME = 1.14 years). The data indicates that the primary error margin for this framework shifted to the early-to-mid adulthood interval (30–40 years), suggesting that resolving subtle individual variances during structural maturity remains a relative challenge for the hybrid architecture.

Senescence and Atrophy (Ages 60–96): In the elderly cohort (Table 4), LST-Net recorded an MAE of 2.77 years, which is comparable to the ScaleDense–Transformer (2.78 years). However, the VQC-based refinement contributed to improved error consistency, as evidenced by a lower mMAE (5.25 years vs. 6.54 years) and a higher correlation coefficient. Although a systematic underestimation (ME = −0.68) was observed—potentially reflecting the heterogeneous nature of late-life brain atrophy—the reduced interval error fluctuations suggest that LST-Net offers a relatively stable mapping for tracking progressive neurodegeneration.

Overall, the performance gains derived from the VQC-based refinement are most prominent during the high-variance stages of childhood and adolescence, remain consistently superior during adulthood, and manifest as enhanced predictive stability in the elderly phase.

### 3.2. Performance Evaluation on the Cross-Site Set

As summarized in Table 5, LST-Net maintained favorable predictive accuracy on the cross-site set, achieving an MAE of 3.93 years and an mMAE of 6.02 years. These results reflect quantitative advantages over the baseline configurations, specifically reducing the MAE by over 5 years compared to standalone ScaleDense (9.07 years) or Transformer (9.43 years) models, which exhibited near-total performance degradation under cross-domain shifts. Notably, while the ScaleDense–Transformer exhibited a pronounced systematic underestimation (ME = −2.74), LST-Net yielded a better-calibrated ME of 0.51. This shift, coupled with a 33.3% reduction in mMAE compared to the fusion baseline, suggests that the VQC-based module may support more consistent feature recalibration. This mechanism not only mitigates the directional offsets typically induced by cross-site transfer but also enhances the model’s resilience against worst-case prediction errors in diverse clinical settings.

The stage-specific analysis in Table 6 reveals that the framework preserves strong transferability in early-life cohorts (0–30 years). However, the MAE peaked in the [40, 50) age bin (6.02 years), indicating that early-to-mid adulthood may be particularly sensitive to the interplay between imaging heterogeneity and the cumulative biological variance inherent in this transitional phase. Despite localized fluctuations, the error stabilized in the older cohorts (50–80 years), confirming a consistent predictive capacity; however, the results for the [80, 90) group (MAE = 4.22) should be interpreted with caution due to the limited sample size (*n* = 12) and the observed increase in negative bias (ME = −4.22). An analysis of the ME across these intervals reveals age-dependent shifts—moving from a slight positive bias in youth to a negative bias during mid-adulthood—reflecting a common regression-toward-the-mean effect. Notably, compared to the severe systematic offsets seen in baseline models (Table 1), LST-Net maintains relatively contained ME fluctuations even across domain-shifted sites, suggesting that while global calibration is significantly improved, localized systematic residuals are still influenced by inherent statistical tendencies. These residuals may be further exacerbated by site-specific data distributions, yet the overall alignment remains superior to classical fusion methods.

**Figure 6:**
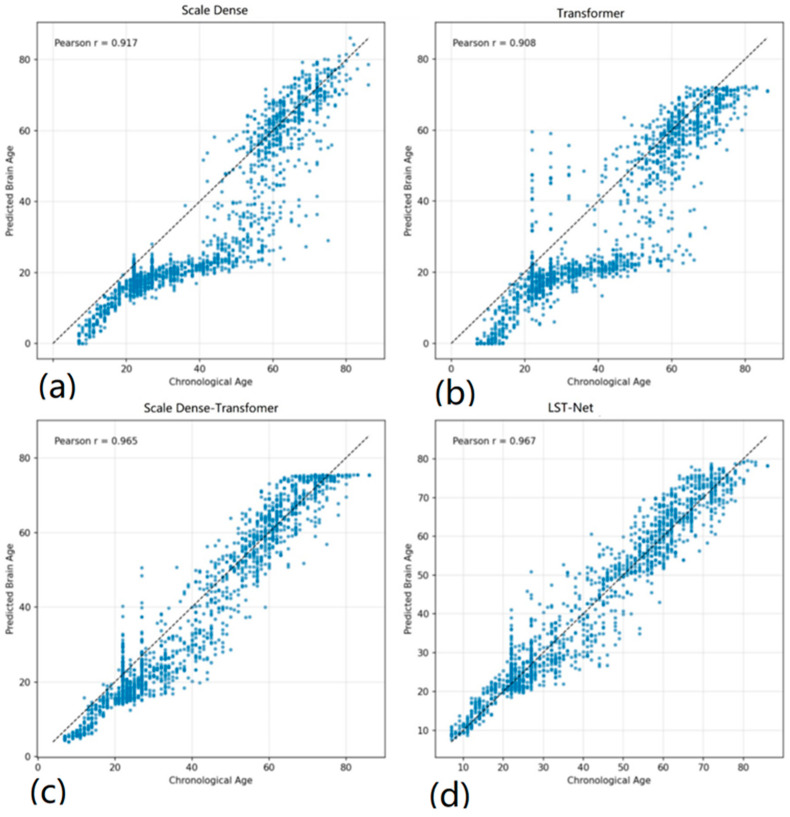
Predicted vs. chronological age scatter plots for the cross-site set. > Panels show results for (**a**) ScaleDense, (**b**) Transformer, (**c**) ScaleDense–Transformer, and (**d**) LST-Net.

Visually, Figure 6 confirms that LST-Net yields tighter clustering around the identity line, suggesting that the integration of the VQC-based refinement fosters a more resilient and noise-robust representation that maintains a stable age-mapping across diverse clinical environments. Specifically, while Scale Dense and Transformer exhibit significant systematic drift and increased dispersion—characterized by a broad scatter band and pronounced underestimation—LST-Net maintains a remarkably consistent and narrow trajectory across the entire lifespan. Notably, the ‘prediction plateaus’ observed in the ScaleDense–Transformer at the older age extremes (above 70 years) are substantially mitigated in LST-Net, which sustains linear alignment even under cross-site domain shifts.

### 3.3. Tracking Stability: Consistency Analysis on the Longitudinal Set

To evaluate the capacity of LST-Net in tracking individual-level brain aging, we analyzed its predictive consistency on the longitudinal set. Unlike cross-sectional evaluations, longitudinal validation emphasizes the temporal progression of structural features within the same subject, offering a robust measure of a model’s sensitivity to subtle neuroanatomical changes over time.

As summarized in Table 7, LST-Net achieved an MdE of −0.09, an MAdE of 4.25, and an mMAdE of 6.34. In terms of average longitudinal deviation, LST-Net achieved favorable longitudinal consistency relative to the standalone ScaleDense and Transformer backbones. Notably, it also demonstrated a more balanced calibration over the ScaleDense–Transformer (MdE = −0.45, MAdE = 4.30). The near-zero MdE (−0.09) indicates that LST-Net provides a precise estimation of temporal brain age shifts, effectively minimizing the systematic bias often encountered in follow-up assessments. While the mMAdE of LST-Net (6.34) was marginally higher than that of the fusion baseline (6.04), this minor trade-off suggests that while the quantum-inspired component is associated with more consistent global trajectory tracking, it may introduce localized sensitivity to atypical aging patterns or subtle scan-to-scan variability.

Furthermore, the consistent performance of LST-Net across both cross-sectional and longitudinal evaluations highlights its robustness as a lifespan-compatible framework. While standalone backbones exhibited significant performance degradation in follow-up settings (MAdE > 5.8 years), LST-Net maintained a stable error profile, with its MdE (−0.09) being substantially lower than its cross-sectional ME observed in earlier stages (Table 2 and Table 3). This convergence toward zero bias in longitudinal tracking—despite the inherent noise in repeat scanning—suggests that the VQC-based feature recalibration effectively filters non-biological variance, preserving the high-fidelity structural cues necessary for monitoring subtle neurodegenerative or developmental trajectories.

### 3.4. Robustness Profiling: Reproducibility on the Repeat-Scan Set

To establish the methodological rigor of the proposed framework, we conducted a test–retest analysis using a dedicated repeat-scan set to assess the reproducibility of age predictions under varying acquisition conditions. As detailed in Table 8, LST-Net achieved favorable reproducibility metrics across all reliability metrics, yielding the highest ICC of 0.994, the lowest mean difference (μ(d) = −0.39), and the most constrained difference dispersion (σ(d) = 1.78).

These results demonstrate that LST-Net consistently outperforms both the standalone backbones and the ScaleDense–Transformer, which recorded an ICC of 0.993 and a higher σ(d) of 2.05. Notably, LST-Net also achieved the minimum scan-wise standard deviation (σ(y′_scan)= 0.59), suggesting that the framework is exceptionally resilient to the subtle acquisition noise and technical artifacts inherent in repeated sessions. The achievement of near-perfect concordance (ICC > 0.99) and the marked reduction in prediction fluctuations indicate that the integration of the VQC-based module enhances feature calibration within a compact latent space.

Collectively, this superior reproducibility confirms that the performance gains of LST-Net are not achieved at the expense of model stability. Such high test–retest reliability is essential for the practical application of brain age as a potential biomarker, ensuring that longitudinal or diagnostic inferences are driven by genuine biological signals rather than methodological inconsistencies.

## 4. Discussion

### 4.1. Mapping the Neurobiological Lifecycle: Non-Linear Trajectory Capture and Structural Heterochronicity

The human brain undergoes profound, non-linear morphological transitions from neonatal development through advanced senescence, characterized by high-dimensional shifts in both localized tissue density and global structural covariance [27,28,29]. Captured across our extensive cohort of 22,271 scans, the LST-Net framework demonstrates a robust capacity to delineate these trajectories, effectively addressing the “boundary problem” inherent in piecewise or age-restricted models. During the first two decades, neuroanatomical maturation involves extensive synaptic pruning and progressive myelination, reflected in region-specific cortical thinning alongside sustained increases in white matter integrity [30,31]. These changes are fundamentally heterochronous, with sensory-motor regions maturing significantly earlier than high-order association cortices [32]. To mirror this biological hierarchy, the ScaleDense component functions as a multi-scale “anatomical lens”; by leveraging dense feature re-use across varying receptive fields, it intrinsically aligns with the localized morphometric “spurts” and high-frequency structural changes that define early neurodevelopment, a capacity reflected in the precision achieved within our adolescent subgroup.

Conversely, late-life aging follows a “last-in, first-out” pattern of neurodegeneration, where prefrontal heteromodal association cortices exhibit the earliest and most significant volume loss [33,34,35]. Physiologically, this progresses from selective pyramidal neuron shrinkage in early senescence [36] to widespread white matter disruption and periventricular leukoaraiosis in advanced stages [37]. Consequently, the brain’s global connectivity shifts from a highly segregated network in young adulthood to a desegregated, less efficient state in later decades. The Transformer module is engineered specifically to address this systemic reorganization; by bypassing the inductive bias of local kernels, it is theoretically designed to model long-range structural covariance patterns and potentially capture distributed age-related structural representations across the brain. Although the observed cross-site generalization behavior is consistent with the utility of this global feature modeling strategy, the precise biological relevance and network-level organization of these representations remain difficult to directly infer from the current experiments. The robustness of this global feature extraction is empirically supported by our cross-site validation results, which demonstrate consistent predictive performance across multiple independent datasets despite significant scanner heterogeneity.

Between these developmental and degenerative poles lies a mid-adulthood plateau—a critical “inflection point” where the rate of structural change fluctuates before accelerating sharply in the seventh decade. These non-linear “braking and accelerating” phases pose significant challenges for conventional regression models. The VQC-inspired module acts as a structured nonlinear feature refinement component at the latent bottleneck, performing parameterized nonlinear transformations of compressed representations within the latent feature space. Through this design, the model may enrich latent representation capacity and facilitate the modeling of complex nonlinear aging-related variations across the lifespan. This additional nonlinear transformation mechanism may partially contribute to the reduced prediction plateau effects observed in LST-Net, potentially by improving the flexibility of feature representations under heterogeneous aging patterns. Consequently, LST-Net achieves a continuous representation of the aging trajectory across the lifespan, enabling smooth estimation of brain age progression. However, a performance decline in the 30–50 age range is still observed, which may reflect inherent challenges in modeling mid-adulthood brain structure, where subtle macrostructural changes and increased inter-individual variability reduce the signal-to-noise ratio compared to early developmental or late degenerative stages. This limitation likely arises from a combination of data characteristics and modeling difficulty under low-amplitude biological signals.

### 4.2. Longitudinal Trajectory Integrity: Sensitivity to Biological Brain Aging

From a methodological and clinical standpoint, the utility of a brain age model extends beyond cross-sectional accuracy to its ability to faithfully track within-subject temporal changes. An ideal longitudinal model should exhibit (i) MdE ≈ 0 and (ii) reduced MAdE, reflecting sensitivity to genuine biological change rather than session-specific variability. Empirically, LST-Net demonstrated a near-zero MdE (−0.09) and a lower MAdE compared to standalone backbone models, indicating favorable calibration and longitudinal sensitivity to intra-individual structural evolution. Notably, this improvement was achieved without a substantial increase in overall variance, although a marginal elevation in mMAdE suggests a nuanced trade-off between sensitivity and robustness. These findings collectively are consistent with the hypothesis that the model reflects meaningful longitudinal variation rather than purely session-specific noise in repeated MRI acquisitions.

Mechanistically, this longitudinal consistency can be partially attributed to the post-bottleneck global integration strategy. By constraining the Transformer module to operate on a compact latent representation, the model shifts from high-dimensional spatial attention to low-dimensional relational recalibration. This design may facilitate aggregation of distributed structural cues into a coherent representation, thereby potentially reducing susceptibility to local voxel-level fluctuations that are common across scanning sessions. In contrast to conventional CNN-based approaches—where predictions may be disproportionately influenced by localized intensity variations—the global attention mechanism may promote cross-region consistency, which is consistent with more stable predictions across time. The precise mechanistic contribution of the VQC-based bottleneck warrants a more nuanced interpretation. It is better understood as a compact non-linear feature transformer operating in a highly compressed latent space. This module may provide an additional nonlinear transformation of latent representations, which could contribute to improved sensitivity to subtle morphological variations that are not linearly separable. However, this interpretation remains speculative, as direct mechanistic evidence is not provided in the present study.

The interpretation of longitudinal performance must be contextualized within the inherent limitations of repeat MRI data. Even under rigorous preprocessing, session effects—including scanner drift, repositioning variability, and physiological noise—introduce a non-negligible error floor. While the high test–retest reliability observed in this study suggests that such effects are reasonably controlled, it remains possible that part of the detected longitudinal variation reflects residual non-biological variance. The slight increase in mMAdE further indicates that sensitivity to subtle changes may come at the cost of increased susceptibility in atypical or low signal-to-noise scenarios. From a translational perspective, the ability to produce stable and biologically aligned longitudinal trajectories is central to the use of brain age as a biomarker. A model that minimizes systematic drift while preserving sensitivity to gradual structural change is better positioned for applications in early disease detection, monitoring of neurodegenerative progression, and evaluation of intervention effects. In this regard, the observed balance between stability and responsiveness in LST-Net represents a potentially meaningful step toward clinically relevant brain age tracking, although further validation is required before clinical translation can be claimed.

### 4.3. Comparative Evaluation with Contemporary Brain Age Models

LST-Net demonstrates competitive performance relative to several contemporary brain-age architectures, achieving a MAE of 2.71 years (Table 9). While direct numerical comparisons in Table 9 should be interpreted with caution—given the systematic variations in cohort composition, preprocessing pipelines, and evaluation protocols across studies—the robustness of LST-Net remains noteworthy. Its performance was validated on an extensive, heterogeneous dataset comprising 22,271 subjects across a 96-year lifespan. The ability of LST-Net to maintain high predictive stability across different data sources, despite significant scanner-related and anatomical variability, underscores the efficacy of the ScaleDense–Transformer architecture in capturing complex, non-linear neuroanatomical trajectories. This reliability in a multi-source data environment, coupled with its competitive benchmarking results, reinforces the potential utility of LST-Net for large-scale clinical screening and longitudinal brain health monitoring.

### 4.4. Constraints and Future Exploration

Despite the promising performance and robustness demonstrated by LST-Net, several limitations must be acknowledged to provide a balanced perspective on its current stage of development.

First, although our study leverages an extensive dataset spanning the full lifespan (0–96 years), the inherent age distribution remains inevitably imbalanced. Specifically, samples at the extreme ends of the spectrum—such as neonates and the oldest-old—are relatively sparse compared to the middle-aged and young-adult cohorts. This is largely because our framework utilizes large-scale public repositories that are heavily concentrated in the mid-life and early-senescence brackets, such as the typical 40–70 age distribution of the UKB, meaning that this statistical imbalance may introduce subtle predictive biases at the chronological extremes. Concurrently, subgroup-stratified analyses were not conducted, as the present work focuses on global performance evaluation under a fixed multi-cohort benchmarking setting rather than detailed stratified interpretation. Second, a pivotal characteristic of LST-Net is its neuroscience-inspired, design-driven philosophy, rather than an optimization-driven paradigm. The ScaleDense–Transformer architecture was specifically engineered to align with the biological priors of brain aging: utilizing ScaleDense for local anatomical features and Transformers for global network reorganizations. While we prioritized the neurobiological plausibility of this “local-global” integration, we did not perform exhaustive hyperparameter tuning or large-scale Neural Architecture Search to push the model to its absolute mathematical limit. Specifically, we did not perform explicit interpretability mapping to fully characterize latent representations, nor did we include predictive uncertainty estimation or fairness evaluation across demographic subgroups. In addition, parameter-matched classical neural network baselines were not exhaustively explored. Third, the current integration of the auxiliary VQC represents an early-stage exploration of quantum-inspired hybrid modeling. Due to the limitations of the current Noisy Intermediate-Scale Quantum era, the VQC component was implemented via classical simulation on a limited number of qubits. While this was sufficient to demonstrate the potential of quantum circuits in capturing high-order non-linear couplings in brain aging, the true computational advantages often associated with quantum hardware—such as potential efficiency gains or enhanced representational expressivity—remain to be fully realized on physical quantum systems. Consequently, the scalability of this quantum bottleneck—such as varying the virtual qubit topologies, optimizing parameterized circuit depths, or evaluating architectural resilience under multiple random initialization seeds—is inherently constrained by the exponential memory overhead required for high-dimensional classical simulation graphs. Furthermore, although the T1-weighted structural MRI was prioritized for its high clinical availability and consistency across multi-site protocols, relying solely on macro-structural information may overlook the subtle functional alterations and microstructural white-matter reorganization that often precede gross anatomical changes. External clinical validation on pathologically diagnosed patient cohorts remains absent, thereby limiting immediate inferences regarding the framework’s real clinical utility and diagnostic biomarker potential in real-world environments.

Looking ahead, future research will focus on the following four primary directions: (1) Moving beyond predictive precision, it is crucial to decode the “black box” of the ScaleDense–Transformer representations. Future studies will integrate advanced attribution techniques, such as SHapley Additive exPlanations (SHAP) or attention-based saliency mapping, to systematically examine which neuroanatomical regions drive the model’s decisions at different life stages. Aligning these learned patterns with established neurodevelopmental and neurodegenerative trajectories will strengthen the neurobiological plausibility of LST-Net. (2) The ultimate value of a brain-age biomarker lies in its “Brain-Age Gap” sensitivity. We plan to extend LST-Net to specific patient populations to assess its utility in detecting accelerated aging and its potential as a surrogate marker for disease progression and treatment response. (3) As quantum hardware evolves, we aim to migrate our VQC module from classical simulators to actual quantum processors to investigate the impact of physical quantum noise on model stability and explore the processing of even higher-dimensional neuroimaging features. (4) Future iterations could incorporate diffusion MRI or fMRI into a unified architecture. Additionally, implementing multi-task learning—simultaneously predicting brain age and classifying cognitive status—could further enhance the robustness of the learned representations and provide a more holistic assessment of brain health.

## 5. Conclusions

In this study, we introduced LST-Net, a design-driven framework for brain age prediction that integrates a ScaleDense backbone with a Transformer-based fusion module and an auxiliary quantum-inspired component. Validated on an extensive dataset of 22,271 subjects across many heterogeneous sources, LST-Net achieved a competitive MAE of 2.71 years. The primary strength of this model lies in its multi-scale architectural design, utilizing ScaleDense for localized anatomical feature extraction and Transformers for global feature integration across distributed representations. Furthermore, the incorporation of VQC provides an additional nonlinear transformation in the compressed latent space, which may contribute to improved modeling of complex age-related structural variation. Crucially, as LST-Net was developed following a design-centric rather than a purely optimization-driven paradigm, its efficacy is rooted in structural logic rather than brute-force parameter tuning. Ultimately, this study balances predictive precision with neurobiological plausibility, providing a promising and scalable tool for longitudinal brain health monitoring and large-scale population screenings, although further external and clinically controlled validation will be necessary before translational applications can be established.

## Figures and Tables

**Table 1:** Benchmarking results of LST-Net and baseline models across the entire internal evaluation set.

Model Name	MAE	ME	mMAE	r	R^2^
Scale Dense	4.33	−2.55	18.53	0.97	0.90
Transformer	5.19	−3.15	22.72	0.96	0.86
ScaleDense–Transformer	3.29	−0.57	15.98	0.98	0.95
LST-Net	2.71	0.13	5.77	0.98	0.97

**Table 2:** Predictive efficacy of various architectures during the neurodevelopmental stage (Ages 0–18) within the internal evaluation set.

Model Name	MAE	ME	mMAE	r	R^2^
Scale Dense	4.27	−4.17	4.80	0.77	−0.66
Transformer	7.28	−6.83	7.69	0.51	−3.45
ScaleDense–Transformer	3.61	−3.37	4.23	0.79	−0.17
LST-Net	1.82	1.10	2.01	0.88	0.62

**Table 3:** Predictive efficacy of various architectures during structural maturity stage (Ages 18–60) within the internal evaluation set.

Model Name	MAE	ME	mMAE	r	R^2^
Scale Dense	5.74	−4.23	12.65	0.93	0.76
Transformer	6.72	−3.94	12.79	0.93	0.70
ScaleDense–Transformer	3.92	−1.30	8.21	0.98	0.91
LST-Net	2.70	1.14	5.77	0.98	0.95

**Table 4:** Predictive efficacy of various architectures during the senescence and atrophy stage (Ages 60–96) within the internal evaluation set.

Model Name	MAE	ME	mMAE	r	R^2^
Scale Dense	3.25	−1.15	4.84	0.72	0.20
Transformer	3.86	−2.28	10.56	0.58	−0.13
ScaleDense–Transformer	2.78	0.20	6.54	0.76	0.52
LST-Net	2.77	−0.68	5.25	0.77	0.52

**Table 5:** Comparative performance and evaluation metrics across models on the cross-site set.

Model Name	MAE	ME	mMAE
Scale Dense	9.07	−7.73	18.38
Transformer	9.43	−7.70	18.06
ScaleDense–Transformer	5.36	−2.74	9.03
LST-Net	3.93	0.51	6.02

**Table 6:** Evaluation of age-specific generalization and robustness for LST-Net using the cross-site evaluation set.

Age Bin	Subjects (n)	MAE	ME
[0, 10)	24	1.92	1.92
[10, 20)	140	2.17	1.63
[20, 30)	366	3.23	1.18
[30, 40)	136	5.41	−1.17
[40, 50)	150	6.02	−1.34
[50, 60)	213	4.11	0.91
[60, 70)	256	4.20	1.52
[70, 80)	127	3.39	−1.22
[80, 90)	12	4.22	−4.22

**Table 7:** Evaluation of Longitudinal Consistency Across Models.

Model Name	MdE	MAdE	mMAdE
Scale Dense	0.07	5.83	10.93
Transformer	−0.13	6.54	10.51
ScaleDense–Transformer	−0.45	4.30	6.04
LST-Net	−0.09	4.25	6.34

**Table 8:** Performance and test–retest reliability metrics of various models on the repeat-scan set.

Model Name	σ(y′_scan)	μ(d)	σ(d)	ICC
Scale Dense	0.61	−0.41	2.55	0.988
Transformer	0.93	−0.48	2.98	0.984
ScaleDense–Transformer	0.67	−0.50	2.05	0.993
LST-Net	0.59	−0.39	1.78	0.994

**Table 9:** Contextual comparison with representative brain-age models.

Reference	Model	Age Range	Dataset(s)	Subjects	MAE (Years)
Besson et al. [38]	Graph CNNs	7–89	11 public datasets	6410	4.58
Cheng et al. [39]	VGGNets	8–80	8 public datasets	3743	4.45
Couvy-Duchesne et al. [40]	Ensemble models	17–90	PAC2019	2640	3.33
He et al. [41]	Lightweight Fully CNNs	8–80	10 public datasets	3054	4.45
Lim et al. [42]	Graph Attention with ResNet	20–70	7 public datasets	2788	2.82
Popescu et al. [43]	UNet	18–90	21 public datasets	4422	9.94
Proposed Method	LST-Net	0–96	17 public datasets (modeling cohort)	22,271	2.71

## Data Availability

This research relies exclusively on publicly accessible neuroimaging repositories, as detailed in Supplementary Materials Table S1. In support of open science and study replication, these platforms provide standardized pathways for researchers to request data access and acquisition, contingent upon adherence to their specific institutional governance and data-use protocols.

## Supplementary Materials

The following supporting information can be downloaded at: https://www.mdpi.com/article/10.3390/brainsci16060581/s1, Figure S1: An overview of the age distribution of all critical datasets in the research. (a) to (e) correspond sequentially to the sex-stratified age distribution of the modeling cohort (*n* = 22,271), the age distribution of the modeling cohort’s training set (*n* = 15,582), validation set (*n* = 3341), internal evaluation set (*n* = 3348), and the age distribution of the cross-site set (*n* = 1422). Table S1: Comprehensive Overview of the Key Characteristics Associated with the Databases and Cohorts Utilized in the Study; Table S2: Structural specifications, input/output dimensions, and parameter allocation across LST-Net.
