# Supplementary material for: A Lightweight ScaleDense–Transformer Framework with Auxiliary Quantum-Inspired Bottleneck Module for Whole-Lifespan Brain Age Prediction
Source: Brain Sci. 2026 May 29;16(6):581. doi: 10.3390/brainsci16060581 (PMC13297227; doi:10.3390/brainsci16060581)
Supplement: Supplementary file 1 [file brainsci-16-00581-s001.zip › brainsci-4304372-supplementary.pdf]

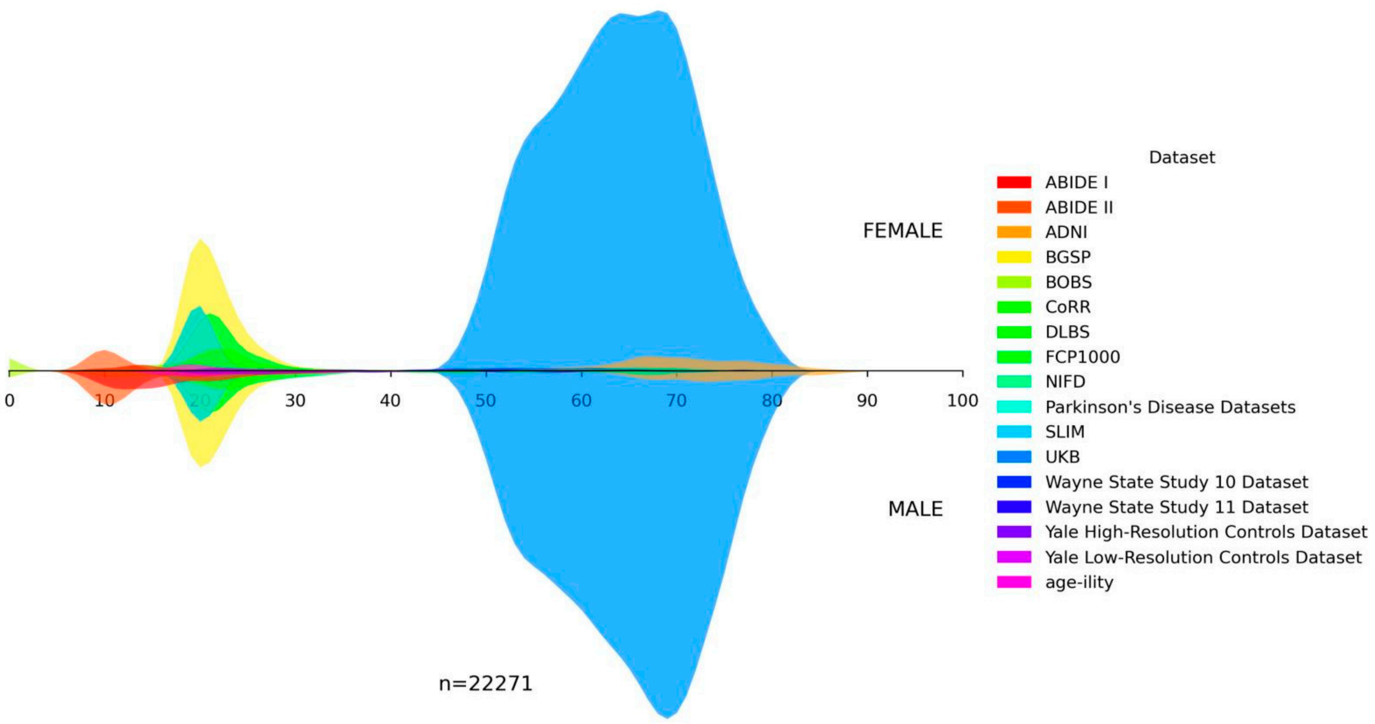
FEMALE
MALE
0
10
20
30
40
50
60
70
80
90
100
n=22271
Dataset
ABIDE I
ABIDE II
ADNI
BGSP
BOBS
CoRR
DLBS
FCP1000
NIFD
Parkinson's Disease Datasets
SLIM
UKB
Wayne State Study 10 Dataset
Wayne State Study 11 Dataset
Yale High-Resolution Controls Dataset
Yale Low-Resolution Controls Dataset
age-ility

(a)

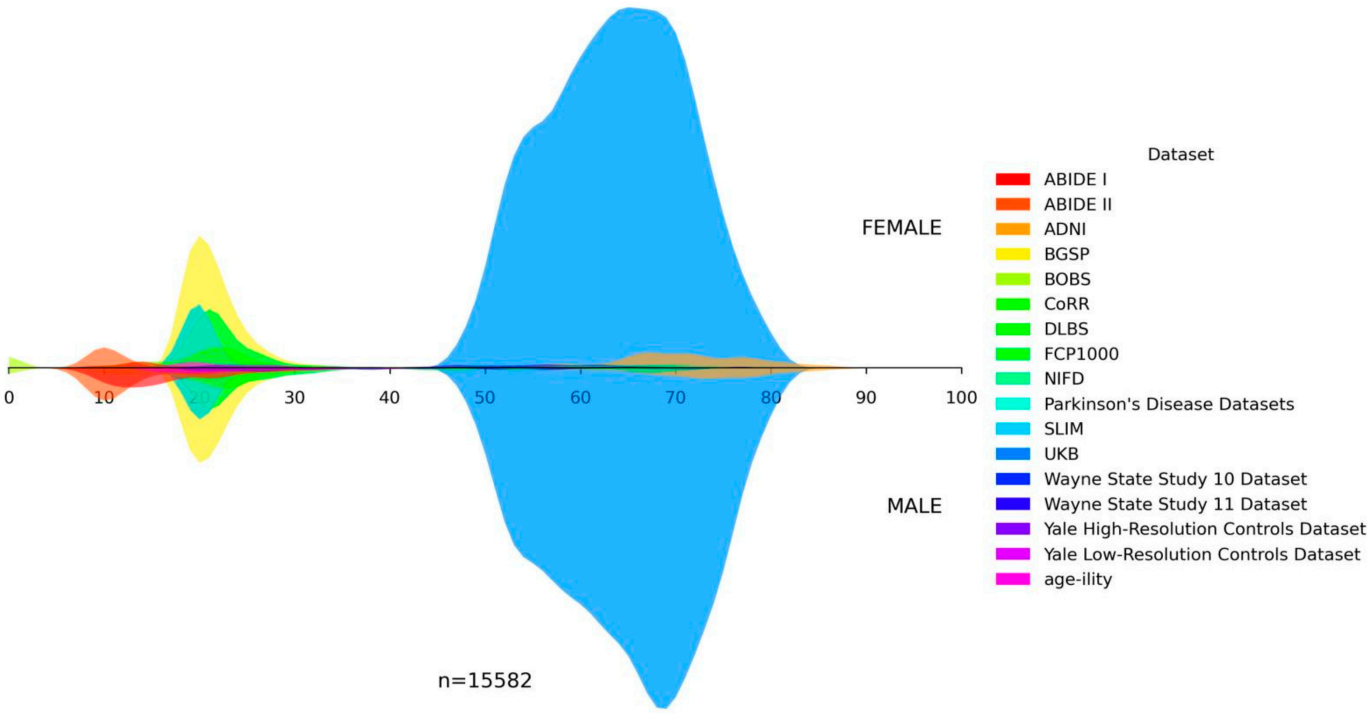
FEMALE
MALE
0
10
20
30
40
50
60
70
80
90
100
n=15582
Dataset
ABIDE I
ABIDE II
ADNI
BGSP
BOBS
CoRR
DLBS
FCP1000
NIFD
Parkinson's Disease Datasets
SLIM
UKB
Wayne State Study 10 Dataset
Wayne State Study 11 Dataset
Yale High-Resolution Controls Dataset
Yale Low-Resolution Controls Dataset
age-ility

(b)

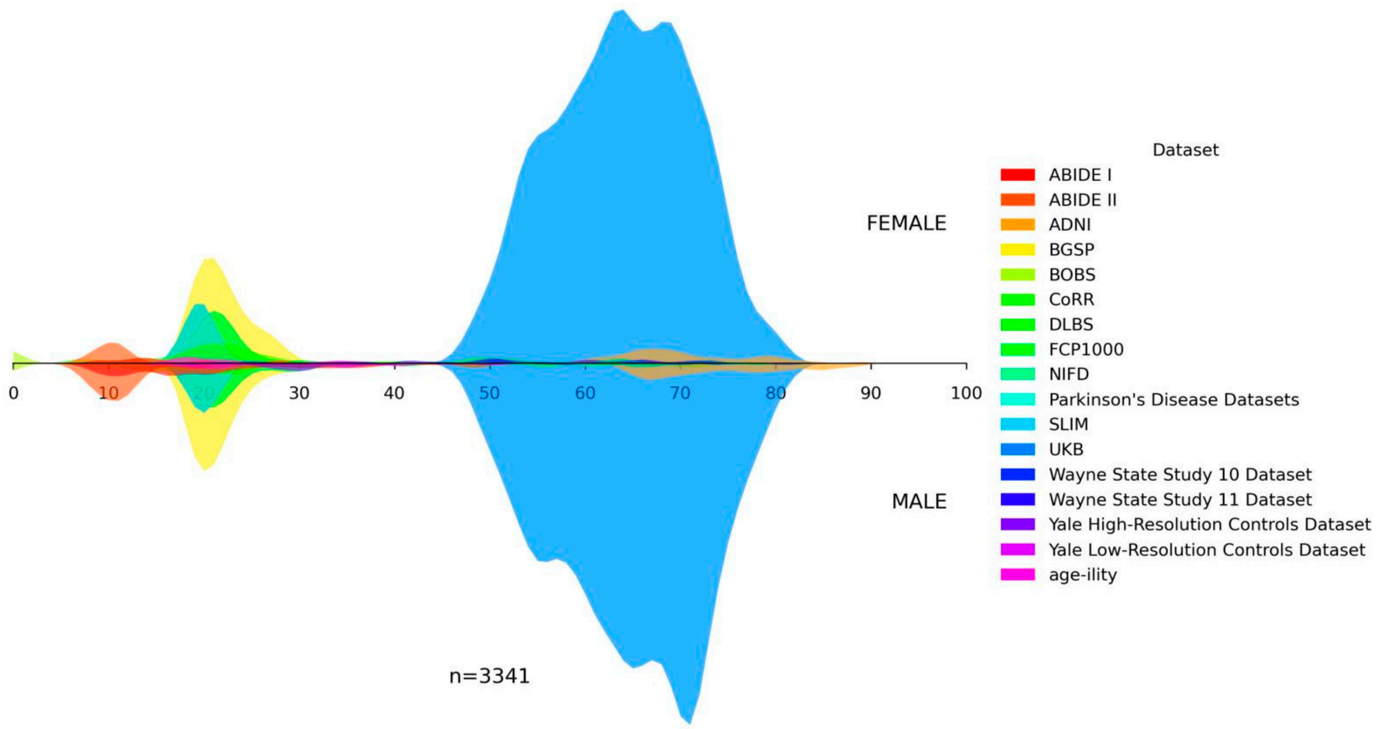
FEMALE
MALE
n=3341
0
10
20
30
40
50
60
70
80
90
100
Dataset
ABIDE I
ABIDE II
ADNI
BGSP
BOBS
CoRR
DLBS
FCP1000
NIFD
Parkinson's Disease Datasets
SLIM
UKB
Wayne State Study 10 Dataset
Wayne State Study 11 Dataset
Yale High-Resolution Controls Dataset
Yale Low-Resolution Controls Dataset
age-ility

(c)

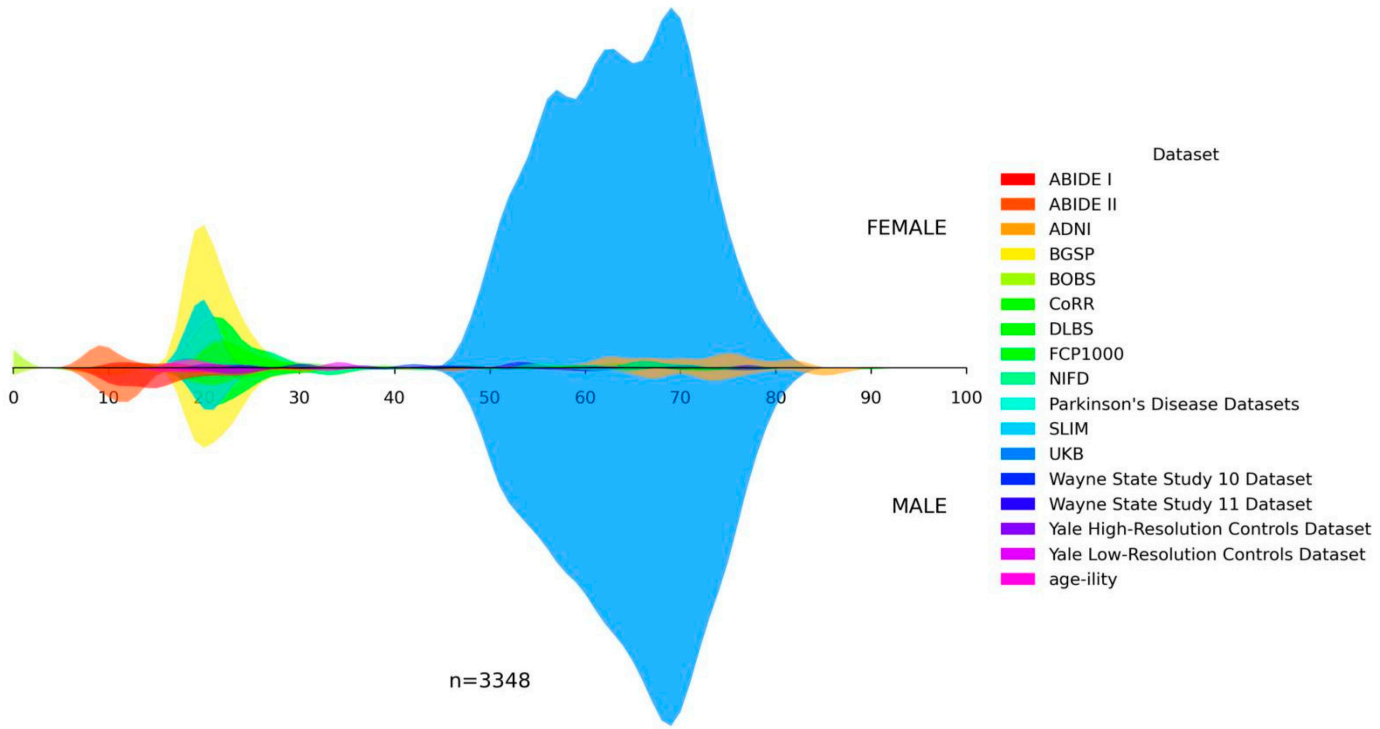

FEMALE
MALE
n=3348
0
10
20
30
40
50
60
70
80
90
100
Dataset
ABIDE I
ABIDE II
ADNI
BGSP
BOBS
CoRR
DLBS
FCP1000
NIFD
Parkinson's Disease Datasets
SLIM
UKB
Wayne State Study 10 Dataset
Wayne State Study 11 Dataset
Yale High-Resolution Controls Dataset
Yale Low-Resolution Controls Dataset
age-ility

(d)

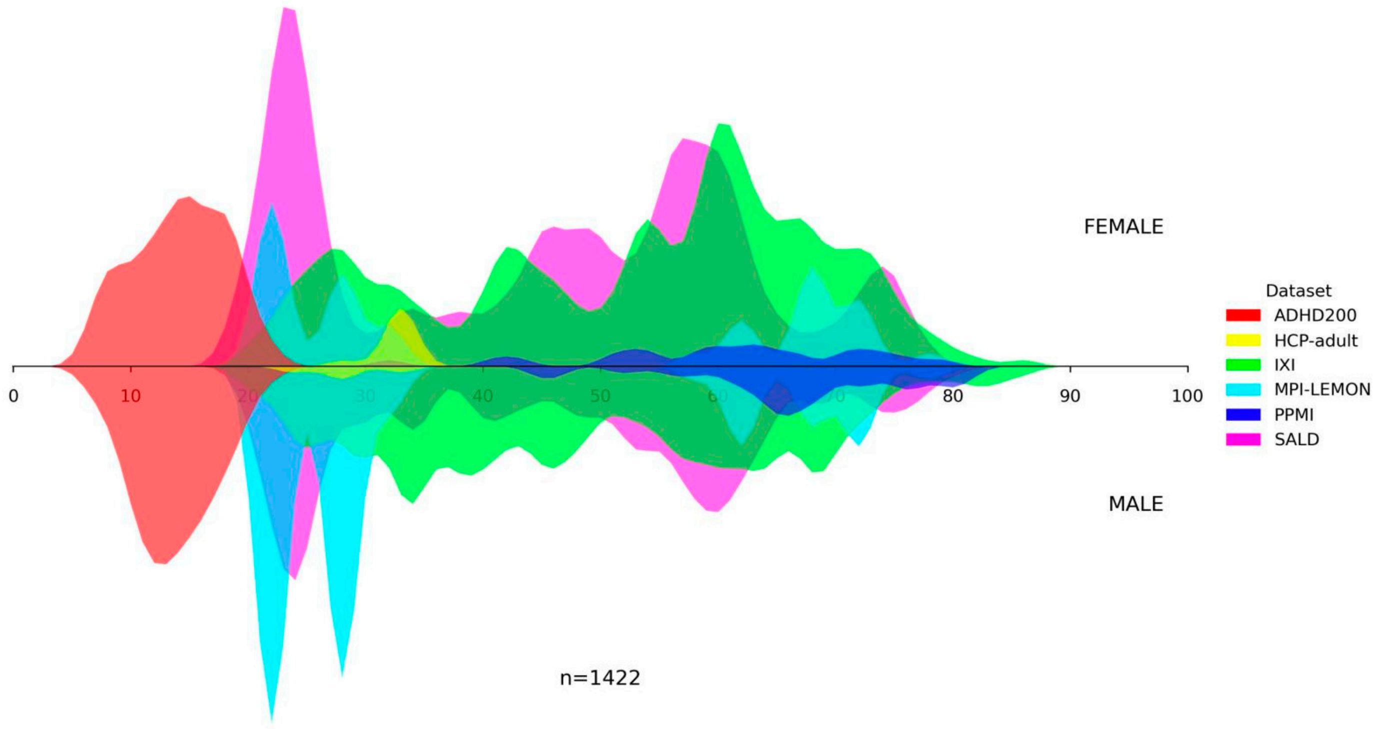

(e)

Figure S1. An overview of the age distribution of all critical datasets in the research. (a) to (e) correspond sequentially to the sex-stratified age distribution of the modeling cohort (n = 22,271), the age distribution of the modeling cohort's training set (n = 15,582), validation set (n = 3,341), internal evaluation set (n = 3,348), and the age distribution of the cross-site set (n = 1,422).

Table S1. Comprehensive Overview of the Key Characteristics Associated with the Databases and Cohorts Utilized in the Study

Dataset	Age Interval	Number of Subjects (n)	Gender Distribution (M/F)	Scanner (Magnetic Field)	Target Function	Additional Function	Web Link
Attention Deficit Hyperactivity Disorder (ADHD200)	7-22	165	81/84	Multi-platform scanners (1.5T, 3T)	Cross-site set		http://fcon_1000.projects.nitrc.org/
Alzheimer's Disease Neuroimaging Initiative (ADNI)	51-95	506	214/ 292	Multi-platform scanners (1.5T, 3T)	Modeling cohort		https://adni.loni.usc.edu/
age-ility	15-35	131	66/65	Siemens (3T)	Modeling cohort		https://www.nitrc.org/projects/age-ility/
Brain Genomics Superstruct Project (BGSP)	19-35	1567	661/ 903	Siemens (3T)	Modeling cohort		https://ida.loni.usc.edu/login.jsp?project=NIFD
Baby Open Brains Repository (BOBS)	0-1	43	14/29	Siemens (3T)	Modeling cohort		https://bobsrepository.readthedocs.io/en/latest/data_access/
Consortium for Reliability and Reproducibility (CoRR)	6-88	1359	680/ 679	Multi-platform scanners (3T)	Repeat-scan set	Modeling cohort	https://doi.org/10.15387/fcp_indi.corr.hnu1
Dallas Lifespan Brain Study(DLBS)	20-90	171	52/119	Philips (3T)	Longitudinal set	Modeling cohort	http://fcon_1000.projects.nitrc.org/indi/retro/dlbs.html
Frontotemporal Lobar Degeneration Neuroimaging Initiative	36-84	136	58/78	Multi-platform scanners (3T)	Modeling cohort		https://ida.loni.usc.edu/login.jsp?project=NIFD

(NIFD)						
Human Connectome Project-adult (HCP-adult)	22-35	85	48/37	Siemens (3T)	Cross-site set	http://www.humanconnectome.org/data/data-use-terms/
Human Connectome Project (HCP-retest)	22-38	29	10/19	Siemens (3T)	Repeat-scan set	http://www.humanconnectome.org/data/data-use-terms/
Information eXtraction from Images(IXI)	20-87	480	210/270	Multi-platform scanners (1.5T, 3T)	Cross-site set	https://www.nitrc.org/projects/ixi_dataset
Max Planck Institut Leipzig Mind-Brain-Body Dataset (MPI-LEMON)	20-80	226	145/81	Siemens (3T)	Cross-site set	http://fcon_1000.projects.nitrc.org/
Parkinson's Disease Datasets	46-82	24	13/11	Siemens (1.5T, 3T)	Modeling cohort	http://fcon_1000.projects.nitrc.org
Parkinson's Progression Markers Initiative(PPMI)	32-81	52	30/22	Siemens (3T)	Cross-site set	https://www.ppmi-info.org/
Southwest University Adult Lifespan (SALD)	19-80	485	183/302	Siemens (3T)	Cross-site set	http://fcon_1000.projects.nitrc.org/indi/retro/sald.html
Southwest University Longitudinal Imaging Multimodal (SLIM)	17-27	573	253/320	Siemens (3T)	Modeling cohort	http://fcon_1000.projects.nitrc.org/
the Autism Brain	6-57	366	298/68	Multi-platform	Modeling	http://fcon_1000.projects.nitrc.or

Imaging Data Exchang I (ABIDE I)				scanners (3T)	cohort		g/
the Autism Brain Imaging Data Exchang II (ABIDE II)	5-64	461	326/ 135	Multi-platform scanners (3T)	Longitudinal set	Modeling cohort / Repeat-scan set	http://fcon_1000.projects.nitrc.or g/
The Open Access Series of Imaging Studies I (OASIS I)	18-96	64	17/47	Siemens (1.5T)	Repeat-scan set		https://sites.wustl.edu/oasisbrai ns/
The Open Access Series of Imaging Studies II (OASIS II)	60-96	24	6/18	Siemens (1.5T)	Repeat-scan set		https://sites.wustl.edu/oasisbrai ns/
UK Biobank(UKB)	45-82	16377	7811/ 8566	Siemens (3T)	Modeling cohort		https://www.ukbiobank.ac.uk/
Wayne State Study 10 Dataset	19-83	111	35/76	Siemens (1.5T)	Modeling cohort		http://fcon_1000.projects.nitrc.or g/indi/retro/wayne_11.html
Wayne State Study 11 Dataset	21-79	181	123/58	Siemens (4T)	Modeling cohort		http://fcon_1000.projects.nitrc.or g/indi/retro/wayne_11.html
Yale High-Resolution Controls Dataset	18-58	120	68/52	Siemens (3T)	Modeling cohort		http://fcon_1000.projects.nitrc.or g
Yale Low-Resolution Controls Dataset	18-66	99	50/49	Siemens (3T)	Modeling cohort		http://fcon_1000.projects.nitrc.or g
Yale Repeat-scan set	27-56	12	6/6	Siemens (3T)	Repeat-scan set		http://fcon_1000.projects.nitrc.or g
1000 Functional Connectomes	7-85	865	384/ 481	Multi-platform scanners	Modeling cohort /	Repeat-scan set/	http://fcon_1000.projects.nitrc.or g/

Project(FCP1000)	(1.5T, 3T, 4T)	Longitudinal set	

Table S2. Structural specifications, input/output dimensions, and parameter allocation across LST-Net

Stage / Module	Core Functional Mechanism	Input Tensor Dimension	Output Tensor Dimension	Parameter Count
Input Layer	Input	1×84×102×84	1×84×102×84	0
Initial Layer	3D Convolution (7×7×7, stride=1, dil=2)	1×84×102×84	8×84×102×84	2,752
ScaleDense Stage 1	Two stacked 3D AC-Blocks + SE Block (r=16)	8×84×102×84	24×42×51×42	13,856
ScaleDense Stage 2	Two stacked 3D AC-Blocks + SE Block (r=16)	24×42×51×42	72×21×25×21	124,704
ScaleDense Stage 3	Two stacked 3D AC-Blocks + SE Block (r=16)	72×21×25×21	216×10×12×10	1,122,336
ScaleDense Stage 4	Two stacked 3D AC-Blocks + SE Block (r=16)	216×10×12×10	648×5×6×5	10,101,024
ScaleDense Stage 5	Two stacked 3D AC-Blocks + SE Block (r=16)	648×5×6×5	1944×2×3×2	90,909,216
Bottleneck Proj.	Global Adaptive Average Pooling + Linear	1944×2×3×2	1944×1×1×1	62,240
VQC Bottleneck	32-8-32 Projection + 2-layer Virtual Qubits	32D Classical Vector	32D Refined Vector	600
Global Transformer	8 Slots×4 Dim, 2 Encoder Layers (h=2)	32D Refined Vector	32D Harmonized Vector	488
Regression Head	Multi-layer Projection + ReLU	32D Harmonized Vector	1D Scalar (Predicted Age)	545
Total Framework				~102 M
